# Contour detection improved by context-adaptive surround suppression

**DOI:** 10.1371/journal.pone.0181792

**Published:** 2017-07-31

**Authors:** Qiang Sang, Biao Cai, Hao Chen

**Affiliations:** 1 College of Information Science & Technology, Chengdu University of Technology, Chengdu, Sichuan, P.R.China; 2 Department of Digital Media Technology, Chengdu University of Technology, Chengdu, Sichuan, China; 3 Department of Computer Science and Technology, Southwest University for Nationalities, Chengdu, Sichuan, China; Chinese Academy of Sciences, CHINA

## Abstract

Recently, many image processing applications have taken advantage of a psychophysical and neurophysiological mechanism, called “surround suppression” to extract object contour from a natural scene. However, these traditional methods often adopt a single suppression model and a fixed input parameter called “inhibition level”, which needs to be manually specified. To overcome these drawbacks, we propose a novel model, called “context-adaptive surround suppression”, which can automatically control the effect of surround suppression according to image local contextual features measured by a surface estimator based on a local linear kernel. Moreover, a dynamic suppression method and its stopping mechanism are introduced to avoid manual intervention. The proposed algorithm is demonstrated and validated by a broad range of experimental results.

## Introduction

Contour extraction is one of the most important tasks in computer vision and pattern recognition. It has been extensively studied in image segmentation, shape matching and motion tracking. The goal of contour extraction is to find meaningful edge points of object contour. But it is very difficult to distinguish the true object boundaries from the confounding non-meaning edges from texture fields, especially in natural images. Some traditional operators can not distinguish the edges generated from texture or objects. However, the human visual system has the mechanism to extract the main contour rapidly and effectively. Recently, the task benefits from a biologically motivated mechanism—receptive field or non-receptive field (RF or Non-RF) that is exhibited by most orientation selective neurons in the primary visual cortex. That influences the perception of groups of edges or lines [1–3]. Levitt J.B. et al. [4] demonstrate that the responses to a stimulus place within a V1 neuron’s receptive field can be either increase or decrease by adding a stimulus in the region surrounding the receptive field. Psychophysical and neurophysiological findings [5–8] have shown that the cortical cell can be taken as a part of an interactional network rather than an isolated element. Namely, the perception of an oriented stimulus can be influenced by the presence of other such stimuli in its neighborhood. In the area of computer version, it is called surround suppression (SS).

**Fig 1:**
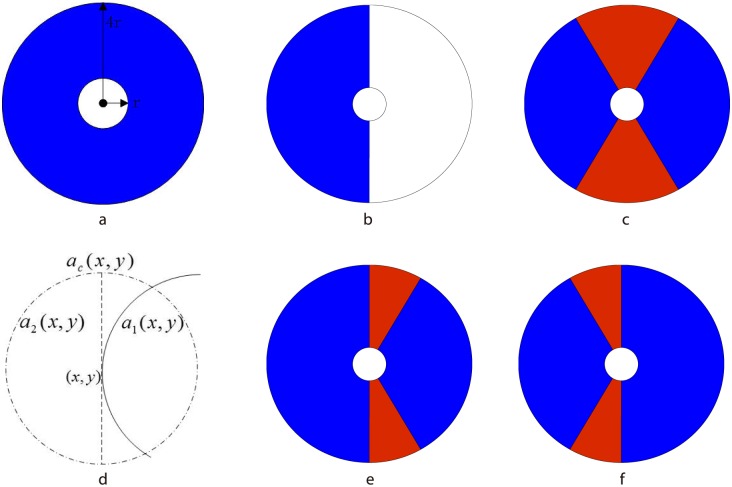
Suppression models. (a)SS, the suppression originates between radius *r*_1_ and *r*_2_ (*r*_2_ = 4*r*_1_). (b)model in [11], the suppression only in one side of SS would be computed. (c)CSS, the blue area produces inhibitory effect and the red area produces excitatory effect. (d)Decomposition of the support of the kernel function. (e)-(f)CASS, the two model in CASS would be adaptively chose according to the local feature of image.

The mechanism has been integrated into some traditional edge detectors. Initially, Grigorescu et al. [9] use the method of non-classical receptive inhibition to effectively suppress surrounding textures and admirably preserve isolated contours by combining Gabor filter and SS shown in Fig 1a. Grigorescu and colleagues [10] combine the Canny detector with SS to extract contour. The methods in [9] and [10] are effective for dense texture areas. Nevertheless, it leads to undesirable, partial self-inhibition of isolated edges and considerable inhibition of texture region boundaries. Papari and colleagues [11] propose to split the inhibition surround into two truncated half-rings oriented along the concerned edge and compute the inhibition term as the minimum of the two weighted averages on these two truncated half-rings as shown in Fig 1b.

Moreover, the stimuli of surround suppression can also enhance the response of V1 neuron when they are aligned with the center to form collinear contextual stimuli, and this called spatial facilitation [12–15]. Tang Q. et al. [16, 17] unify spatial facilitation and surround inhibition to present a compound surround suppression (CSS) shown in Fig 1c, where the red region denotes the excitatory area Ω_*E*_ and the blue region denotes the inhibitory area Ω_*I*_. The stimuli imposed on the excitatory region would enhance the response of the center point. Chi Z. et al. [18] also adopt a similar model to distinguish the side and end subregions of nCRF that wording in different manners. Chi Z. et al [19] propose a model based on the theory of steerable filters for the inhibition term and introduces a method to combine the binary edge maps obtained by different inhibition levels. In a recent study [20], the authors propose an orientation-selective inhibition model, which combines isotropic and anisotropic inhibition mechanisms into a single model. Papari G. et al [21] introduce a multi-scale scheme that multiple parameters of suppression levels are adopted and the set of edge points is merged to obtain the final object contour. A multi-scale integration based contour extraction model inspired by the inhibitory and disinhibitory interactions between the classical receptive field and the non-classical receptive field is presented [22].

However, these previous works just utilize the single suppression model and lack the adaptability to image local contextual features which results in suppression of weak object boundaries or retention of strong texture edges. In this paper, we propose a context-adaptive surround suppression (CASS) model, which can simultaneously suppress strong texture edge and preserve weak contour edge. Meanwhile, because most of the traditional methods utilize the single inhibition level, which often weakens the performance of surround suppression, the multi-level inhibition [21] is proposed to resolve this problem. But the method needs manual intervention and is time consuming. Based on the theory of surround suppression, this paper also proposes a nonlinear dynamic suppression method (DSM) and its stopping mechanism to set suppression levels without manual intervening and adaptively control the suppression strength in the local region. Experiments show that the new method achieves good quality and improvement in efficiency relative to the traditional methods.

The present paper is organized as follows: Section 2 gives the description of previous work and the proposed method in this paper, followed by a number of experiments and validations using real natural image examples. Discussions are drawn in Section 4.

## Methods

### surround suppression

Grigorescu et al. [9] introduce an operator that includes surround inhibition for enhanced contour detection. This method relies on the premise that if edges are close to each other, they are likely to be a texture. On the contrary, isolated edges are likely to be true contours. They extend a gradient magnitude operator with a term which takes into account the context influence of the surroundings of a given point.

Firstly, a scale-dependent gradient is computed. Let ∇_*x*_ *f*_*σ*_(*x*,*y*) and ∇_*y*_ *f*_*σ*_(*x*, *y*) be the *x*− and *y*–components of the scale-dependent gradient:

$$\begin{array}{ccc} \nabla_{x}f_{\sigma }\left(x,y\right) & = & \left(f*\frac{\partial g_{\sigma }}{\partial x}\right)\left(x,y\right),\\ \nabla_{y}f_{\sigma }\left(x,y\right) & = & \left(f*\frac{\partial g_{\sigma }}{\partial y}\right)\left(x,y\right), \end{array}$$ (1)

where *g*_*σ*_ is a two-variate Gaussian function. The scale-dependent gradient magnitude *M*_*σ*_(*x*, *y*) is given by:

$$\begin{array}{c} M_{\sigma }\left(x,y\right)=\sqrt{(\nabla_{x}f_{\sigma }{\left(x,y)\right)}^{2}+{\left(\nabla_{y}f_{\sigma }\left(x,y\right)\right)}^{2}}. \end{array}$$ (2)

And, two weighting functions are adopted to simulate the SS. One is the distance weighting function. Let *DoG*_*σ*_(*x*, *y*) be the following difference of two Gaussian function:

$$\begin{array}{c} DoG_{\sigma }\left(x,y\right)=\frac{1}{2\pi {\left(4\sigma \right)}^{2}}exp\left(-\frac{x^{2}+y^{2}}{2{\left(4\sigma \right)}^{2}}\right)-\frac{1}{2\pi \sigma^{2}}exp\left(-\frac{x^{2}+y^{2}}{2\sigma^{2}}\right). \end{array}$$ (3)

The distance weighting function is defined as follows:

$$\begin{array}{c} w\left(x,y\right)=\frac{H(DoG_{\sigma }\left(x,y\right))}{∥H\left(DoG_{\sigma }\right){∥}_{1}}, \end{array}$$ (4)

where $H(z)=\{\begin{array}{cc} z, & z>=0\\ 0, & z<0 \end{array}$ and ‖ ⋅ ‖_1_ is the *L*_1_ norm. The inhibition term is computed for each image point and is a weighted sum of the values of the gradient in the suppression surround of the concerned point. The distance between the center point and its neighborhood point is taken into account by the weighting function *w*(*x*, *y*). The other is the orientation weighting function:

$$\begin{array}{c} \Delta (x,y,x-u,y-v)=|cos(\theta (x,y)-\theta (x-u,y-v))|, \end{array}$$ (5)

where (*u*, *v*) is the offset between the center point (x, y) and its neighborhood point (x-u, y-v). If the gradient orientations of points (x, y) and (x-u, y-v) are identical, the orientation weighting factor takes a maximum. The value of the factor decreases with the angle difference *θ*(*x*, *y*) − *θ*(*x* − *u*, *y* − *v*) and reaches a minimum when the two gradient orientations are orthogonal.

For each image point (*x*, *y*), the term *s*(*x*, *y*) is defined as the following weighted sum of the gradient magnitude values *M*_*σ*_(*x* − *u*, *y* − *v*) in the suppression surround of the point:

$$\begin{array}{c} s\left(x,y\right)=\int \int_{\Omega }M_{\sigma }\left(x-u,y-v\right)w\left(u,v\right)\Delta \left(x,y,x-u,y-v\right)dudv, \end{array}$$ (6)

where Ω is the image suppression domain (the blue area in Fig 1a). The two weighting factors (*w*(*u*, *v*) and Δ(*x*, *y*, *x* − *u*, *y* − *v*)) take into account the distance and gradient orientation difference, respectively. An operator *E*(*x*, *y*) takes as its inputs the gradient magnitude *M*(*x*, *y*) and the suppression term *s*(*x*, *y*):

$$\begin{array}{c} E\left(x,y\right)=H(M_{\sigma }\left(x,y\right)-\alpha s\left(x,y\right)), \end{array}$$ (7)

where *α* controls the strength of the suppression. After that the non-maximal suppression and double-threshold are adopted to trace the contour.

### Context-adaptive surround suppression

The main objective of this work is to extract contour in natural images while eliminating the non-meaning texture edges and enhancing the object boundaries as much as possible. To this end, we propose a context-adaptive contour extraction algorithm via a surface estimator based on local linear kernel in this section.

A 2-D regression model for discontinuous surface estimation is:

$$\begin{array}{c} Z_{i}=m\left(X_{i},Y_{i}\right)+\varepsilon_{i},i=1,...,n,\varepsilon_{i}∼\left(0,\sigma^{2}\right), \end{array}$$ (8)

where *m* is the true images with *n* pixels. *Z*_*i*_ *s* represent the observations. (*X*_*i*_, *Y*_*i*_)*s* are pixel points and *ε*_*i*_ *s* represent the zero-mean Gaussian noise with variance *σ*^2^. Local linear kernel smoothing estimates 2-D regression surface by minimizing the weighted mean square error within local area:

$$\begin{array}{cc} \left({\hat{a}}_{c}\left(x,y\right),{\hat{a}}_{c,x}\left(x,y\right),{\hat{a}}_{c,y}\left(x,y\right)\right)=argmin_{a,b,c}\sum_{i=1}^{n} & \\ {\left(Z_{i}-a-b\left(X_{i}-x\right)-c\left(Y_{i}-y\right)\right)}^{2}\cdot K_{B}\left(\left(X_{i}-x\right),\left(Y_{i}-y\right)\right), \end{array}$$ (9)

where ${\hat{a}}_{c}\left(x,y\right)$, ${\hat{a}}_{c,x}\left(x,y\right)$ and ${\hat{a}}_{c,y}\left(x,y\right)$ estimate *a*, *b* and *c* respectively which determine the local regression surface.

*K*(*x*, *y*) is a kernel function defined by:

$$\begin{array}{c} K\left(x,y\right)=(\left(exp\left(-\left(x^{2}+y^{2}\right)/2\right)-exp\left(-0.5\right)\right)/\left(2\pi -3\pi exp\left(-0.5\right)\right)), \end{array}$$ (10)

which has a support {(*x*, *y*):*x*^2^ + *y*^2^ ≤ 1} and is the truncated 2-D Gaussian density function. $K_{B}\left(x,y\right)=\frac{1}{\left|B\right|}K\left(B^{-1}\cdot {\left(x,y\right)}^{t}\right)$. B is a 2 × 2 global bandwidth matrix *diag*(*h*, *h*) and *h* is the scale of the support.

In [23], the support of the kernel function is decomposed as *sc*_1_ and *sc*_2_ along a direction perpendicular to the gradient direction, shown as in Fig 1d. Two one-sided local linear kernel estimations *a*_1_(*x*, *y*) and *a*_2_(*x*, *y*) are computed respectively according to Formula (9). The quality of the three estimators ${\hat{a}}_{r}\left(r=c,1,2\right)$ can be analyzed by the Weighted Residual Mean Squares (WRMS):

$$\begin{array}{c} WRMS_{r}\left(x,y\right)=\frac{1}{\sum_{i}K_{b}\left(i\right)}\sum_{i}{\left[Z_{i}-{\hat{a}}_{r}\left(x,y\right)-{\hat{a}}_{r,x}\left(x,y\right)\left(X_{i}-x\right)-{\hat{a}}_{r,y}\left(x,y\right)\left(Y_{i}-y\right)\right]}^{2}\cdot K_{b}\left(i\right). \end{array}$$ (11)

For point (*x*, *y*), *diff*(*x*, *y*) is defined as follows:

$$\begin{array}{c} diff\left(x,y\right)=max\{WRMS_{c}\left(x,y\right)-WRMS_{1}\left(x,y\right),WRMS_{c}\left(x,y\right)-WRMS_{2}\left(x,y\right)\}. \end{array}$$ (12)

When the neighborhoods of point (*x*, *y*) are homogeneous, *diff*(*x*, *y*) is close to zero because the values of all the WRMS’s are close to the noise variance *σ*^2^ [24]. In the interior of uniform texture region, the value of *diff*(*x*, *y*) is also closed to zero because the value of each WRMS is almost equal. Thus, the strength of surround suppression should decrease with WRMS growth. In CASS, a weighting function *w*_*t*_ is defined as follows:

$$\begin{array}{c} w_{t}\left(r_{m}\left(x,y\right)\right)=exp\left(\frac{r_{m}{\left(x,y\right)}^{2}}{2\sigma_{m}^{2}}\right), \end{array}$$ (13)

where *r*_*m*_(*x*, *y*) = *min*(*WRMS*_1_(*x*, *y*), *WRMS*_2_(*x*, *y*), *WRMS*_*c*_(*x*, *y*)) and *σ*_*m*_ establishes the decrease degree with *r*_*m*_(*x*, *y*). Here we do not consider spatial facilitation but suppression inhibition.

On the other hand, when point (*x*, *y*) is close to an edge segment, the value of *diff*(*x*, *y*) is relatively large because *WRMS*_1_ or *WRMS*_2_ is less than *WRMS*_*c*_. These edge points would locate at the one-sided region whose WRMS is more. Based on that, we propose the improved suppression model which can adaptively determine the excitatory region according to the direction of edge as shown in Fig 1e–1f. The distance weighting functions can be refined as *w*_*i*_ and *w*_*e*_ respectively:

$$\begin{array}{c} w_{i}\left(x,y\right)=\left\{ \begin{array}{cc} w(x,y), & \left(x,y\right)\in c_{k}\cap \left(x,y\right)\in \Omega_{I};\\ 0, & otherwise, \end{array}\right. \end{array}$$ (14)

$$\begin{array}{c} w_{e}\left(x,y\right)=w\left(x,y\right)-w_{i}\left(x,y\right), \end{array}$$ (15)

where *c*_*k*_(*k*|(*WRMS*_*k*_ = *max*(*WRMS*_1_, *WRMS*_2_))is one of the two one-sided regions. Thus the inhibition term *s*_*i*_ and the excitatory term *s*_*e*_ are as follows:

$$\begin{array}{c} s_{i}\left(x,y\right)=\int \int_{\Omega }M_{\sigma }\left(x-u,y-v\right)w_{i}\left(u,v\right)\Delta \left(x,y,x-u,y-v\right)dudv, \end{array}$$ (16)

$$\begin{array}{c} s_{e}\left(x,y\right)=\int \int_{\Omega }M_{\sigma }\left(x-u,y-v\right)w_{e}\left(u,v\right)\Delta \left(x,y,x-u,y-v\right)dudv. \end{array}$$ (17)

The adaptive suppression term *s*_*a*_(*x*, *y*) is defined as follows:

$$\begin{array}{c} s_{a}\left(x,y\right)=\left\{ \begin{array}{cc} w_{t}s\left(x,y\right), & diff(x,y)\leq Thr;\\ s_{i}\left(x,y\right)-s_{e}\left(x,y\right), & diff(x,y)>Thr, \end{array}\right. \end{array}$$ (18)

where *Thr* is the threshold used to determine whether it has a boundary in the neighborhood region of point (*x*, *y*). In this study we experimentally set *Thr* = 30.

The Formula (7) is redefined as follows:

$$\begin{array}{c} E=H(M_{\sigma }\left(x,y\right)-\alpha s_{a}\left(x,y\right)). \end{array}$$ (19)

### Dynamic suppression level

In the previous section, we propose an improved suppression term. Subsequently, what we need to do is to suppress the gradient magnitude intensity of a texture region by the suppression term. The previous methods based on surround suppression almost adopt the single inhibition level, namely the *α* in formula (7) is a constant. But it is difficult to find an appropriate value of *α*. When the inhibition level is set with a high value, many weak edges are suppressed. On the contrary, some intensive textures would remain. As to the multi-level inhibition method, it can resolve the question to a certain extent, but it needs manual intervention and needs to combine different binary maps produced by different suppression levels. In this section, we propose a novel dynamic suppression method, which can adaptively determine the strength of surround suppression. For the intensive texture edge, the suppression effect can continuously works. For the faint contour edge, it would cease quickly. According to [9] and [10], the theory that surround suppression as a biology visual property can be used in edge extraction is mainly based on an assumption that there are many stimuli points (high gradient magnitude points) around the point in the texture area and there are few stimuli points around the point in the edge. Similarly, we make a further assumption that the probability that a point belongs to a texture region is higher if its suppression effect is greater and vice versa. Here a partial differential equation is adopted to simulate the time course changes:

$$\begin{array}{c} \frac{d}{dt}E\left(x,y\right)=H\left[E\left(x,y\right)-\alpha \left(s_{a}\left(x,y\right),Thr\right)s_{a}\left(x,y\right)\right]. \end{array}$$ (20)

Here,

$$\begin{array}{c} \left\{ \begin{array}{c} E\left(x,y\right)=H[M_{\sigma }\left(x,y\right)-\alpha \left(s_{a}\left(x,y\right),Thr\right)s_{a}\left(x,y\right)],t=1;\\ E^{t+1}\left(x,y\right)=H\left[E^{t}\left(x,y\right)-\alpha \left(s_{a^{\prime }}^{t}\left(x,y\right),Thr\right)s_{a^{\prime }}^{t}\left(x,y\right)\right],t>1, \end{array}\right. \end{array}$$ (21)

where the term *M*_*σ*_(*x* − *u*, *y* − *v*) in Formulas (6), (16) and (17) would be replaced with *E*^*t*−1^(*x* − *u*, *y* − *v*) when computing $s_{a^{\prime }}^{t}\left(x,y\right)$.

If the suppression intensity of a point is small relative to its gradient magnitude, the point is almost not influenced by its neighborhood points and vice versa. So, the dynamic mechanism can adjust the suppression intensity according to different image features. For the object contour edge, the suppression intensity that the edge point imposes on its neighborhood points is more than that the neighborhood points impose on the edge point. The suppression strength between the edge point and neighborhood point is not equal. Thus, the suppression strength of the edge point would become weaker and weaker and eventually vanish. For the texture edge, its suppression strength and its neighborhood points’ are almost the same. So, the mutual suppression process would not stop until their gradient magnitudes are reduced to zero. When starting the iterative process, gradient magnitude is used to initialize image response. After that, the image surround suppression response of t times is taken as the input value of t + 1 times. The stopping criterion is: the number of iterations is set a constant number that can experience enough contextual interactions; the iteration would keep on work till most of points are free from the suppression effect. The parameter *α* is set with 0.1 and iteration numbers are 10 to 30. Finally, the non-maximal suppression and double-threshold are used to trace the object contour from the gradient magnitude map. And a quantile *p* is used to compute the high threshold and low threshold.

Thus, the algorithm proposed in this paper can be listed as follows:

**Algorithm 1** Framework of context-adaptive contour detection algorithm.

1: **for** each *point*(*x*, *y*)∈*imagef*(*x*, *y*) **do**

2:  compute the gradient magnitude *M*_*σ*_(*x*, *y*);

3:  compute the *diff*(*x*, *y*) from Eq (12);

4: **end for**

5: **while** No convergence or *iteration* < *Num*_*max*_ **do**

6:  **for** each *point*(*x*, *y*) **do**

7:   **if** *diff*(*x*, *y*)<*Thr* **then**

8:    compute suppression term *s*_*a*_ = *w*_*t*_ *s*(*x*, *y*) from Eqs (6) and (13);

9:   **else**

10:    compute suppression term *s*_*a*_ = *s*_*i*_ − *s*_*e*_ from Eqs (16) and (17);

11:   **end if**

12:   update gradient magnitude *M*_*σ*_(*x*, *y*) = *E* from Eq (19);

13:  **end for**

14: **end while**

15: compute binary image *b*(*x*, *y*) from *M*_*σ*_(*x*, *y*) with the non-maximal suppression and double-threshold;

16: **return** *b*(*x*, *y*)

## Results

In this section, we present some experimental results. The experimental data—40 natural images used in this paper are from [11]. Because the ground truth is given, performance evaluation is carried out by comparing detected contours with the ground truth contours. All the results have been generated with the same values of the input parameter. From the results, we can see that the proposed method outperforms all the others according to suppression of undesired texture and better preservation of low contrast contours.

**Fig 2:**
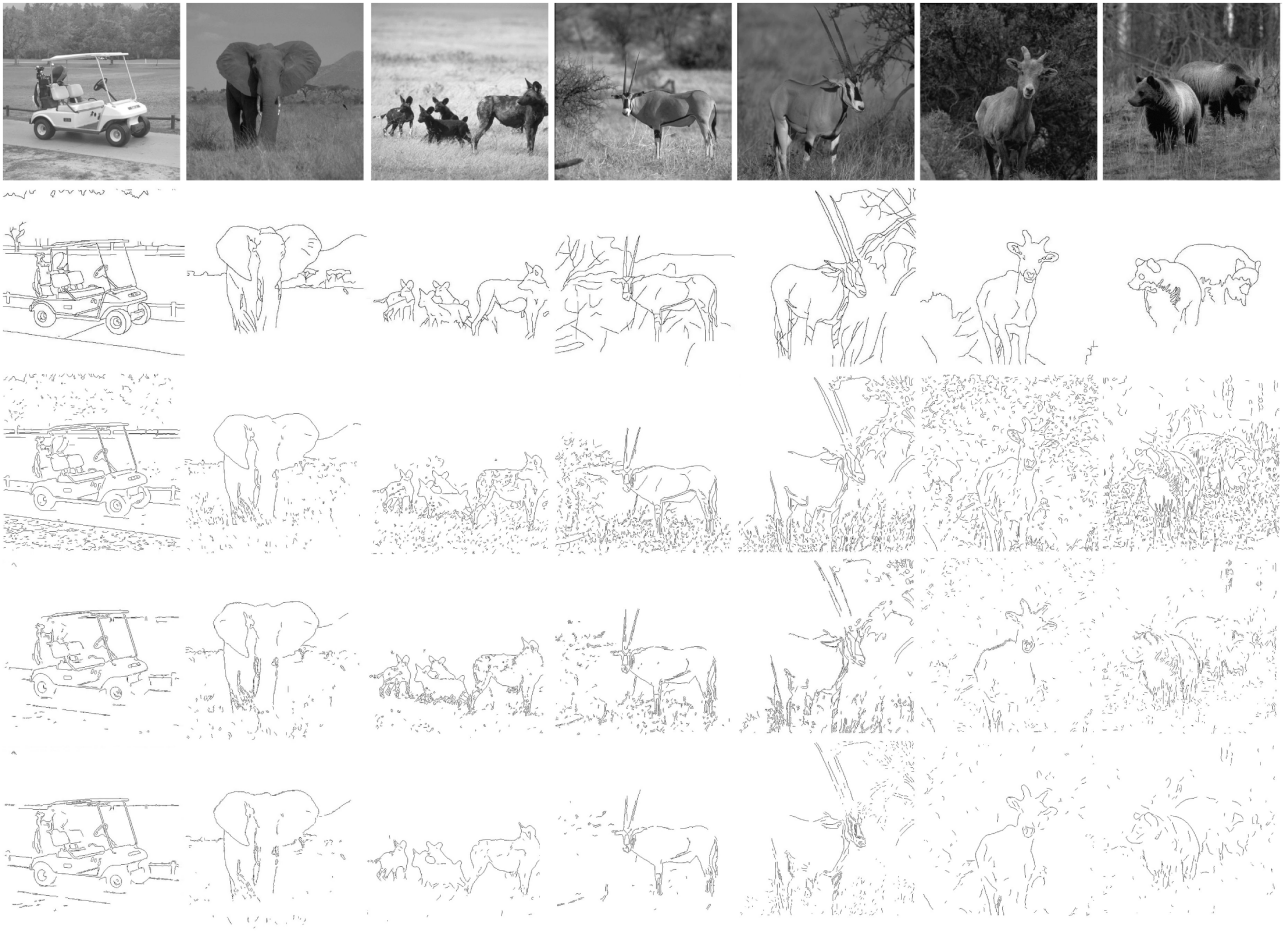
Comparison of contour detection results for different models. From the first to last row: input images, ground truth contour maps, the best results of surround suppression model, the best results of compound surround suppression model, the best results of present algorithm (CASS + DSM). From the first to last col:Golfcart,Elephant2,Hyena,gazelle2,gazelle,goat3,bear.

The proposed method is applied to extract salient contours as shown in Fig 2. Two classical algorithms based on surround suppression [10] and [16] are used to compare with the proposed method in this paper, which combines the adaptive model with the new dynamic suppression method. The best results of contour extraction on seven test images are shown, in which the first and second rows show the input images and the corresponding ground truth contour images, respectively. The third and fourth rows show the best results of classical SS and CSS algorithms, respectively. The last row shows the best results of our method. From Fig 2 we can clearly find that our method eliminates more texture and trivial edge fragments while preserving the boundaries of embedded objects.

To quantify the achieved performance improvement, we use the evaluation criteria proposed by Grigorescu et al. [9]. Let *E*_*GT*_ and *B*_*GT*_ be the sets of edge pixels and background pixels of the ground truth edge image, respectively, and *E*_*D*_ and *B*_*D*_ be the sets of the operated-detected edge images, respectively. The set of correctly detected edge pixels is *E* = *E*_*D*_ ⋂ *E*_*GT*_, false negatives are given by the set *E*_*FP*_ = *E*_*GT*_ ⋂ *B*_*D*_, and the false positives are given by the set *E*_*FN*_ = *E*_*D*_ ⋂ *B*_*GT*_. The percentage of correctly detected edges pixels is:

$$\begin{array}{c} P=\frac{card(E)}{card\left(E\right)+card\left(E_{FP}\right)+card\left(E_{FN}\right)}, \end{array}$$ (22)

where card(x) denotes the number of elements of set *x*. The percentages of false negatives and false positives are *e*_*fn*_ = *card*(*E*_*FN*_)/*card*(*GT*) and *e*_*fp*_ = *card*(*E*_*GT*_)/*card*(*E*), respectively. The means and variances of P, averaged over the 40 images in the data set [11], are plotted.

The scale factor *σ* and quantile *p* are two important parameters in contour extraction algorithm. The *σ* decides the size of surround suppression filter. A higher value of *σ* indicates a higher suppression. And the *p* decides the values of high threshold and low threshold in double-threshold. A higher value of *p* indicates that more image points would be detected as contour points. Then the performances of different algorithms are verified in term of the parameters *σ* and *p* respectively.

**Fig 3:**
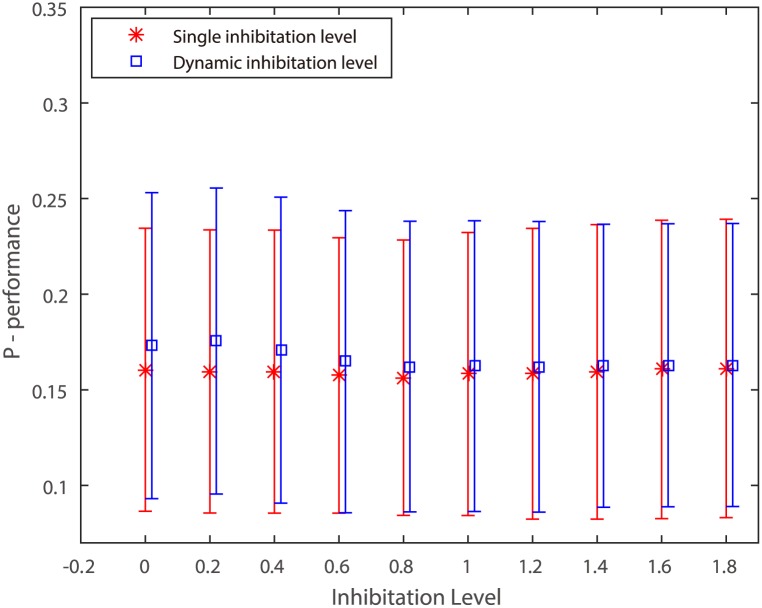
Performance *P* of the single inhibitation level and the dynamic inhibitation level. The single inhibitation and dynamic inhibitation are initialized by *α* levels from 0.01 to 1.8 (step = 0.1). The number of iterations of dynamic suppression is 10.

To show the performance of dynamic inhibitation level, the value of *P* is plotted for single inhibitation level versus dynamic inhibitation level in Fig 3 with the same values of *p* and *σ*. And the values of inhibitation levels *α* are set from 0.01 to 1.81 (step = 0.2). And the number of iteration is 10. The traditional SS model is taken as inhibitation term. As we see, the new inhibitation method outperforms the single inhibitation method when the *α* is set with small value. Because the large value of *α* would lead to over-suppress. Moreover, the best performance of dynamic inhibitation level is better than the single inhibitation level.

**Fig 4:**
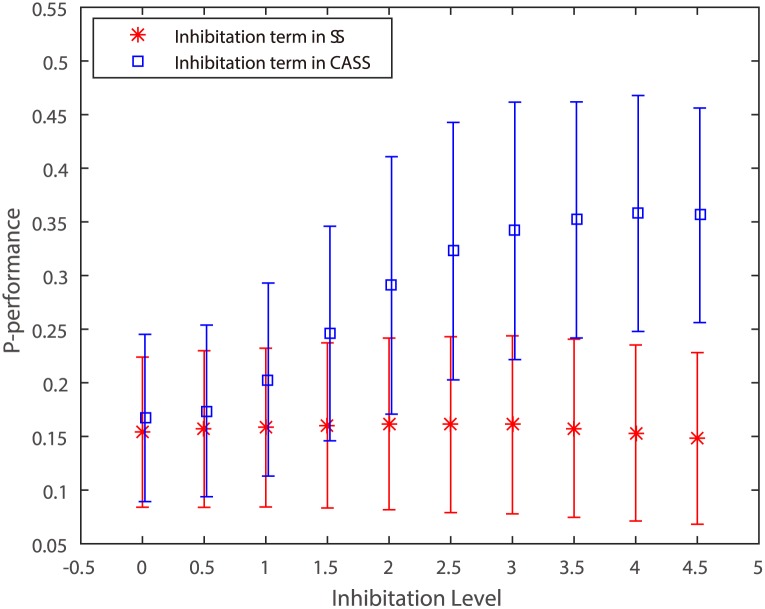
Performance *P* of the tradition inhibition term and the new inhibition term proposed. The inhibitation level *α* is set from 0.01 to 4.51 (step = 0.5).

Next, the average values of *P* are plotted for the traditional inhibitation term in SS versus the inhibitation term in CASS in Fig 4. Here the inhibitation level *α* is set from 0.01 to 4.51 (step = 0.5) and the values of *p* and *σ* are set with 0.2 and 1.0. The inhibitation term proposed here outperforms the traditional one for all values of *α*.

**Fig 5:**
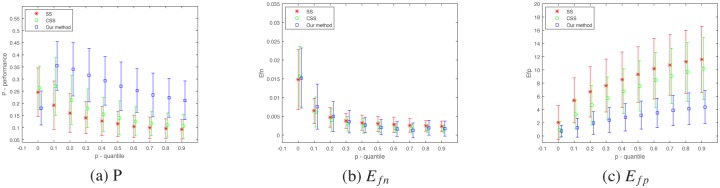
Performance of the SS model, CSS model and our method for different quantile *p*. (a)Performance *P*. (b) *E*_*fn*_. (c) *E*_*fp*_. The values of quantile *p* is set from 0.01 to 0.91 (step = 0.1) and the *σ* is all set with 1.0.

**Fig 6:**
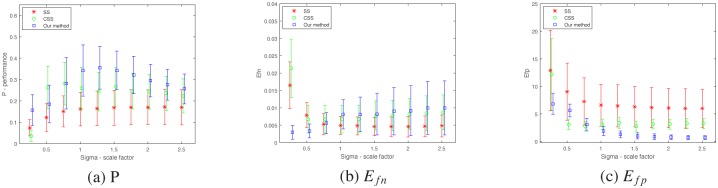
Performance of the SS model, CSS model and our methods for different *σ*. (a)Performance *P*. (b) *E*_*fn*_. (c) *E*_*fp*_. The values of *σ* is set from 0.25 to 2.5 (step = 0.25) and the quantile *p* is all set with 0.2.

The value of *P* is a combinational reflection of texture suppressing and contour retaining. Separately, a smaller *e*_*fn*_ indicates a better retaining of object contour and a lower *e*_*fp*_ means a better suppression of texture. They reflect the different respects of contour extraction that suppression texture and retaining contour are two conflicting task that need to be balanced. The statistical performance *P*, *E*_*fn*_ and *E*_*fp*_ are conducted with different values of *p* and *σ* respectively and plotted in Figs 5 and 6. The algorithms [10] and [16] are used to compare with our proposal method. In Fig 5, the values of *p* are set from 0.01 to 0.91 (step = 0.1) and the *σ* is set with fixed value 1.0. In Fig 6, the values of *σ* are set from 0.25 to 2.5 (step = 0.25) and the *p* is set with fixed value 0.2. The value of *α* is set with 0.1 and the number of iteration is 20 in our method. The *α* is set with 2.0 in the others algorithms. The statistical results show that our method are all better than the previous methods respectively. *E*_*fp*_ indicate a consistent better performance of our model. As to *E*_*fn*_, it is better when the value of *σ* is small. It becomes slightly worse while *σ* increases. So *E*_*fp*_ contributes more for the improvement of performance. That indicates that our method can suppress texture more efficiently.

**Fig 7:**
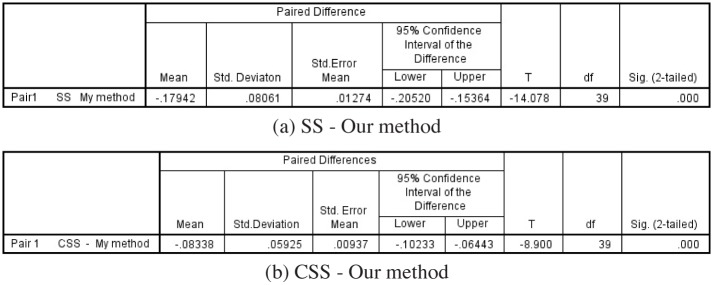
The results of paired t-tests. (a)SS model and our methods. (b)CSS model and our method. The values of *σ* is set with 1.0 and the quantile *p* is set with 0.2.

Finally, a paired t-test of comparing the performance of different models is shown in Fig 7 where Fig 7(a) shows the result of SS and our method and Fig 7(b) is the result of CSS model and our method. The parameters *p* and *σ* are set with 0.2 and 1.0 for all the models. The probabilities of paired t-test are all less than 0.05, which indicates that the performance of our algorithm is definitely improved.

In conclusion, our method can improve the performance of contour extraction of nature images and both the new context-adaptive model and the dynamic suppression method contribute to the improvement of algorithm performance.

## Discussion

Contour is a key feature widely used in pattern recognition and computer vision. However, contour extraction from cluttered scenes is challenging work. To this end, we introduce a simple and efficient context-adaptive surround suppression modal inspired by neural interactions in a primary visual cortex, which combines inhibition and facilitatory effects. Local image features analyzed by a surface estimator is adopted to built adaptively surround model. The main contribution of this work lies in the application of surround suppression and surface feature estimation to contour extraction of the natural scene, which provides piecewise detection operator allows the incorporation of contextual information that improves the performance of algorithm. The proposed method can enhance inhibition of texture region and weaken suppression of contour region. Moreover, a dynamic iteration scheme is proposed in order to avoid manually setting the suppression level parameter, which is different from the traditional multi-scale methods [21]. Experience results have demonstrated the effectiveness of the proposed method in comparison to previous methods.

Despite its advantage, the proposed model still leaves some future research to be done. The contour extraction based on surround suppression mainly takes advantage the low-level visual feature so that it is difficult to distinguish dense contour edge from texture edge. For example, the contour edges of a tree are erased while suppressing the texture edges of grass in the gazelle2 experiment in Fig 3. However, the former belongs to the contour and the latter belongs to the texture according to the ground-true. In our future research, we plan to combine the high-level and low-level visual feature to make the algorithm “analyze” itself. In addition, the iteration numbers of algorithm would be influenced by the parameters of suppression level and suppression strength. When the value of such parameters’ granularity is small, the number of iterations would exceed the traditional methods. When the value is large, our algorithm would degenerate into the constant suppression level. How to control adaptively the granularity of suppression level is also on the agenda for the future.

## Supporting information

S1 Supporting Information The granted permission for the Fig 2. (PNG)
