# Supplementary figures and images for: Contour detection improved by context-adaptive surround suppression
Source: PLoS One. 2017 Jul 31;12(7):e0181792. doi: 10.1371/journal.pone.0181792 (PMC5536361; doi:10.1371/journal.pone.0181792)

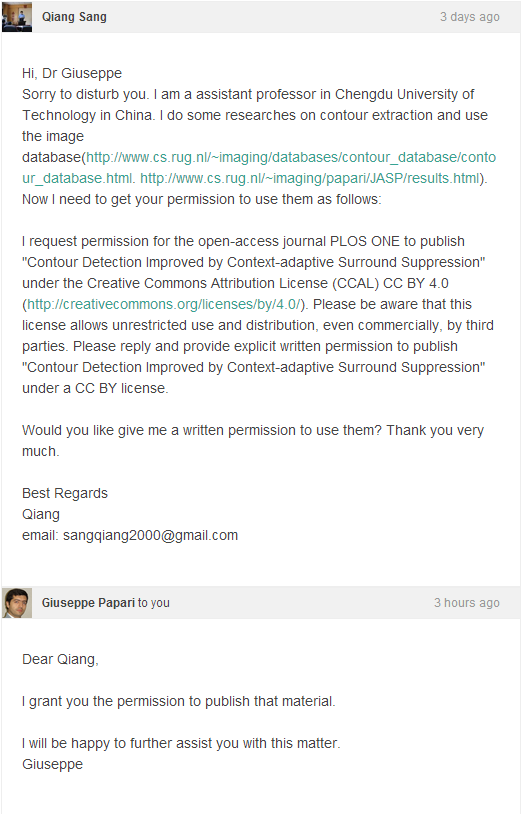

Supplement: S1 Supporting Information — (PNG) [file pone.0181792.s001.png]
